# TRIM37 contributes to malignant outcomes and CDDP resistance in gastric cancer

**DOI:** 10.7150/jca.47577

**Published:** 2021-01-01

**Authors:** Keiji Nishibeppu, Shuhei Komatsu, Jun Kiuchi, Takuma Kishimoto, Yusuke Takashima, Katsutoshi Shoda, Tomohiro Arita, Toshiyuki Kosuga, Hirotaka Konishi, Atsushi Shiozaki, Takeshi Kubota, Kazuma Okamoto, Hitoshi Fujiwara, Hitoshi Tsuda, Eigo Otsuji

**Affiliations:** 1 Division of Digestive Surgery, Department of Surgery, Kyoto Prefectural University of Medicine, 465 Kajii-cho, Kawaramachihirokoji, Kamigyo-ku, Kyoto, Japan.; 2 Department of Pathology, National Cancer Center Hospital, Tokyo, Japan.; 3 Department of Basic Pathology, National Defense Medical College, Tokorozawa, Japan.

**Keywords:** TRIM37, prognosis, proliferation, chemoresistance, gastric cancer

## Abstract

**Background:** TRIM37 (Tripartite Motif Containing 37) is an E3 ubiquitin ligase for histone H2A and inhibits transcription in several genes. However, it is not known whether it plays a role in gastric cancer (GC). In this study, we tested whether TRIM37 acts as a cancer-promoting factor by being overexpressed in GC.

**Methods:** We analyzed GC cell lines and 124 primary tumors, which were curatively resected in our hospital between 2001 and 2003.

**Results:** Overexpression of the TRIM37 protein was detected in almost all GC cell lines and GC samples (76 out of 124 cases) and was significantly correlated with lymphatic and venous invasion, advanced T-Stage, N-Stage, histology and high recurrence rate. Patients with TRIM37 overexpressing tumors had a worse survival rate than those with non-expressing tumors (*P*=0.0057). Moreover, TRIM37 positivity was identified as an independent factor predicting worse outcomes (*P*=0.018, Hazard ratio 3.41). The apoptotic cell analysis showed that the knockdown of TRIM37 increased apoptosis in comparison with the control. In TRIM37 overexpressing GC cells, knockdown of TRIM37 suppressed the migration and invasion.

**Conclusions:** TRIM37 plays a crucial role in tumor malignant potential through its overexpression and highlight its usefulness as a prognostic factor and potential therapeutic target in GC.

## Introduction

Gastric cancer (GC) is one of the leading causes of death from cancer worldwide 1. Although surgical techniques, perioperative chemotherapy regimens, and perioperative management have greatly improved, GC remains one of the most common types of cancer and constitutes a global health-problem 2, 3. Although accumulating evidence shows that several genetic alterations can cause tumor progression and tumorigenesis in GC 4, some therapeutic targets have been identified 5, such as gene amplification of *MET* and *ERBB2*; mutations in *TP53*, *APC*, and *E-cadherin* 6-8; *K-ras* 9 and hypermethylation of *p16* 10, 11; oncogenic activation of *β-catenin*; and inactivation of the mismatch-repair gene *hMLH1*, which is associated with microsatellite instability 12. However, in clinical settings, relatively few genes have been identified as diagnostic biomarkers and therapeutic targets for this cancer 13, 14. Therefore, we aimed to identify clinical biomarkers and therapeutic molecular targets for GC.

Tripartite motif-containing 37 (TRIM37) has been identified as an oncogenic H2A ubiquitin ligase. 15 Ubiquitination of histone H2A is one of the most representative epigenetic markers and is associated with the functions of Polycomb complexes, which play a role in gene silencing and have essential functions during cellular differentiation and development 16-19. The correlation between Polycomb complexes and cellular differentiation indicates that TRIM37 could play an important role in tumorigenesis. The overexpression of TRIM37 has been reported in several malignancies, including gastric cancer, esophageal cancer 20, pancreatic cancer 21, colorectal cancer 22, lung cancer 23, 24, breast cancer 15, and osteosarcoma 25. Chen et al. reported that TRIM37 promoted cell invasion and metastasis in GC 26.

In addition, TRIM37 is located at the critical region of 17q23, in which genomic high-copy amplification has been reported to contribute to the progression of several types of cancer, including prostate cancer 27, germ cell 28, glioblastoma 29, 30, head and neck squamous cell carcinoma 31, as well as GC 32-34. However, to date, there have been few reports on the molecular function of TRIM37, its contribution to gastric carcinogenesis, and its clinical and prognostic significance in patients with GC.

In this study, we wished to investigate the effects of TRIM37 overexpression and activation in GC. Consequently, we demonstrated that TRIM37 was frequently overexpressed in GC cell lines and primary GCs, and the overexpression of TRIM37 was identified as an independent factor associated with a poor prognosis. We also demonstrated that knockdown of *TRIM37* expression in TRIM37-overexpressing GC cells suppressed cell proliferation, migration, invasion, and chemoresistance to cisplatin (CDDP). Our results provided evidence that TRIM37 could be an important molecular marker for determining the malignant properties of tumors and could be a promising therapeutic target in patients with GC.

## Materials and Methods

### Cell lines and primary tissue samples

A total of seven GC cell lines (KATO-III, NUGC4, HGC27, MKN7, MKN28, MKN45, and MKN74 cells) and a fibroblast cell line (WI-38) were used in this study. All cell lines were purchased from RIKEN BioResource Center Cell Bank (Tsukuba, Japan), where the cell lines were authenticated by short tandem repeat (STR) profiling prior to distribution. HGC27 cells were cultured in Dulbecco's Minimum Essential Medium (DMEM). All other cells were cultured in Roswell Park Memorial Institute (RPMI)-1640 medium (Nacalai, Japan). All media were supplemented with 100 mL/L fetal bovine serum (FBS) (Corning, USA). All cell lines were cultured in a humidified incubator at 37°C with 5% CO_2_. Paraffin-embedded primary GC tissue samples were collected from 124 consecutive GC patients who had undergone curative gastrectomy at the Division of Digestive Surgery, Department of Surgery, Kyoto Prefectural University of Medicine (Kyoto, Japan) between 2001 and 2003. Paraffin blocks are stored at room temperature in the dark and we stained the sliced specimens within two weeks. Relevant clinical and survival data were obtained for all patients. All experimental methods were carried out in accordance with relevant guidelines and regulations. Written informed consent was obtained from all patients to use their tissue specimens for research purposes, and the study was approved by the institutional review boards of Kyoto Prefectural University of Medicine. (ERB-C-67) None of the patients underwent endoscopic mucosal resection, palliative resection, preoperative chemotherapy, or radiotherapy, and none of them had synchronous or metachronous multiple cancers in other organs. Disease clinical and pathological stages were defined in accordance with the International Union against Cancer tumor-lymph node-metastases (TNM) classification 35.

### Quantitative RT-PCR

Total RNA was extracted from cell lines using an RNeasy Mini Kit (Qiagen, Valencia, CA, USA). The reverse transcription reaction was performed using a TaqMan MicroRNA Reverse Transcription Kit (Applied Biosystems). The total RNA of normal organs was purchased from Takara Bio Inc., Shiga, Japan (Human Total RNA Master Panel II (Cat. No. 636643). The abundance of mRNA was measured by qPCR using a StepOnePlus PCR system (Applied Biosystems), and cycle threshold (Ct) values were calculated with StepOne Software v2.0 (Applied Biosystems), using TaqMan Gene Expression Assays (Hs01116337_m1 for *TRIM37*; Applied Biosystems) according to the manufacturer's instructions. The results of gene expression were calculated as a ratio between *TRIM37* and an internal reference gene (Hs01060665_g1 for β-actin; Applied Biosystems) that provided a normalization factor for the amount of RNA isolated from a specimen. This assay was performed in triplicate for each sample.

### Western blotting

Anti-TRIM37 rabbit polyclonal antibodies (SLRRAVDPGENSRSKGDC) were purchased from Sigma Aldrich (Tokyo, Japan), anti-ACTB antibodies were purchased from Santa Cruz Biotechnology (Santa Cruz, CA, USA), and anti-ZEB1 antibodies and anti-E-cadherin antibodies were purchased from Cell Signaling Technology (USA). Cells were lysed and their proteins were extracted using M-PER® Mammalian Protein Extraction Reagent (Thermo Scientific, USA).

### Loss-of-function by siRNA and cell growth analysis

For loss-of-function analysis by the knockdown of endogenous gene expression, each of the siRNAs targeting *TRIM37* (sense: GCACCAGUUGCGGUCUUGUAGUAAG, antisense: CUUACUACAAGACCGCAACUGGUGC, Sigma, Tokyo, Japan) or *Luciferase* (*Luc*) 5'-CGUACGCGGAAUACUUCGA-3' (Sigma, Tokyo, Japan) was transfected into cells (10 nmol/L) using Lipofectamine RNAiMAX (Invitrogen Corporation, Carlsbad, CA, USA) according to the manufacturer's instructions. The knockdown of a target gene was confirmed by western blotting.

### Proliferation assay and cell cycle analysis

For measurements of cell growth, the number of viable cells at various time points post-transfection was assessed by a colorimetric water-soluble tetrazolium salt assay (Cell Counting Kit-8; Dojindo Laboratories, Kumamoto, Japan). The cell cycle position was evaluated 72 h post-transfection by fluorescence-activated cell sorting (FACS), as described elsewhere 36.

### Apoptotic cell analysis

At 72 h post-transfection, siRNA-transfected cells were harvested and stained with fluorescein isothiocyanate-conjugated annexin V and phosphatidylinositol using an Annexin V Kit (Beckman Coulter, Brea, CA). A Becton Dickinson Accuri™ C6 flow cytometer was used to analyze the proportion of apoptotic cells.

To assess the chemoresistance of GC cell lines to CDDP and paclitaxel (PTX), NUGC4 (wild-type TP53) and MKN7 (mutant TP53) that had been transfected with siRNA-TRIM37 and its control were plated onto 6-well plates (NUGC4, 3 × 10^4^ cells; MKN7, 5 × 10^4^ cells per well) and incubated overnight under normal culture conditions. The cells were then incubated with CDDP (8 μM) and PTX (0.1 μM). At 48 h after the addition of the anticancer drug, apoptotic cell analysis was performed as mentioned above.

### Transwell migration and invasion assays

Transwell migration and invasion assays were carried out in 24-well modified Transwell Boyden chambers (BD Transduction, Franklin Lakes, NJ, USA). The upper surface of 6.4-mm-diameter filters with 8-µm pores was precoated with (invasion assay) or without (migration assay) Matrigel (BD Transduction). The siRNA transfectants (8 × 10^5^ cells per well) were seeded into the upper chamber with serum-free medium. Complete growth-medium was added to the lower well of each chamber. The transfectants were incubated for 22 h and then migrated or invasive cells on the lower surface of the filters were fixed and stained with Diff-Quik stain (Sysmex, Kobe, Japan). The stained cell nuclei were counted directly in triplicate, as described elsewhere 37-39.

### Immunohistochemistry

Primary tumor samples were fixed with 10% formaldehyde in phosphate buffered saline (PBS) and embedded in paraffin. Paraffin blocks are stored at room temperature in the dark and we stained the sliced specimens within two weeks. A horseradish peroxidase (HRP) staining method was used. Briefly, following deparaffinization, antigen retrieval was performed by heating the samples in 10 mmol/L citrate buffer (pH 9.0) at 95°C for 60 min. Endogenous peroxidases were quenched by incubating the sections in 3% H_2_O_2_ for 20 min. Following treatment with Block Ace (Dainippon Sumitomo Pharmaceutical, Osaka, Japan) for 30 min at room temperature, the sections were further incubated at room temperature for 1 h with anti-TRIM37 antibodies (1:1000). PBS was used for all dilutions and washings. Bound primary antibodies were detected using the EnVision™+ Horseradish Peroxidase System (EnVision+ Dual Link System-HRP; Dako North America, Inc., Carpinteria, CA, USA). HRP labeling was visualized using color development with diaminobenzidine tetrahydrochloride. Slides were counterstained with Mayer's hematoxylin.

For the scoring of TRIM37 expression, the intensity was evaluated for each case (the intensity scores were: 0 = negative, 1 = weak, 2 = moderate, 3 = strong). The expression of TRIM37 was graded as high expression (intensity score ≥ 2 of tumor cells showing immunopositivity), or low expression (intensity score ≤ 1 of tumor cells showing immunopositivity) under high-powered (×200) microscopy 40.

### Statistical analysis

Clinicopathological categorical variables pertaining to the corresponding patients were analyzed for significance using the Chi-square test or Fisher's exact test. Differences in non-categorical variables among subgroups were tested using the non-parametric Mann-Whitney U-test. For the analysis of survival, Kaplan-Meier survival curves were constructed for groups based on univariate predictors, and differences between the groups were tested with the log-rank test. Univariate and multivariate survival analyses were performed using the likelihood ratio test of the stratified Cox proportional hazards model. Differences were assessed with a two-sided test and were considered significant at the *P* < 0.05 level.

## Results

### Overexpression of TRIM37 in gastric cancer cell lines

**Figure 1:**
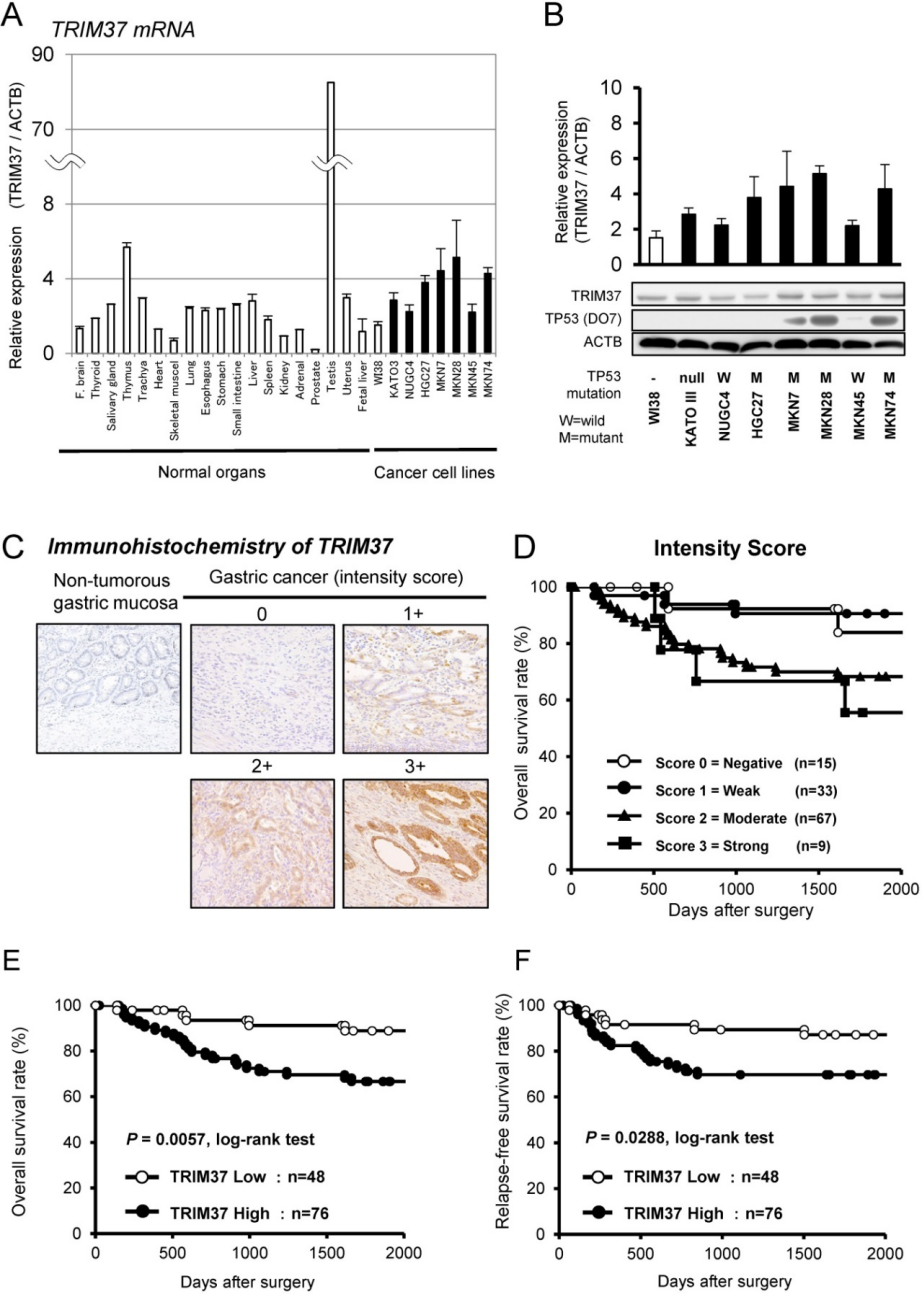
** Overexpression of TRIM37 in gastric cancer. A.** Expression of *TRIM37* mRNA in seven GC cell lines compared with cells from healthy organs plus the fibroblast cell line WI-38.** B.** Expression of TRIM37 in seven GC cell lines compared with the fibroblast cell line WI-38. The level of TRIM37 mRNA determined by quantitative RT-PCR in a panel of GC cell lines. The results shown are means ± SD (bars). Black bars represent cell lines, in which up-regulation of TRIM37 mRNA expression was observed and compared with that in WI-38. In addition, the status of the *TP53* mutation in each cancer cell line was evaluated by western blotting. The status of a *TP53* mutation was positively associated with the reported status of a *TP53* mutation in the database (http://p53.free.fr/index.html, W: wild-type *TP*53, M: mutant *TP53*). Note that among TP53-mutated GC cell lines, KATO-III and HGC27 cells have a p53 gene deletion and a frameshift mutation, respectively. **C.** Specific immunostaining of TRIM37 in a representative primary tumor sample. Based on this result, the intensity scores for TRIM37-staining were determined as follows: 0 = negative, 1 = weak, 2 = moderate, 3 = strong. Kaplan-Meier plots depending on the scores of the intensity of specific immunostaining of TRIM37. **D**. The log-rank test was used for statistical analysis; *P* < 0.05 was considered to be statistically significant. The expression of TRIM37 was graded as being high expression (intensity score ≥ 2 for tumor cells showing immunopositivity) or low expression (intensity score ≤ 1 for tumor cells showing immunopositivity). **E.F.** Overall survival and relapse-free survival rates of patients with GC (as determined by Kaplan-Meier plots), depending on the intensity scores of TRIM37 expression.

Quantitative RT-PCR analysis was performed to test whether *TRIM37* was overexpressed in GC cell lines compared with cells from healthy organs (**Figure 1A**). TRIM37 mRNA overexpression was observed in GC cell lines and the testis compared with healthy organs and the fibroblast cell line WI-38, suggesting that this gene is a cancer-testis antigen (Figure S1 A). TRIM37 protein expression was shown with the mRNA expression in GC cell lines by western blotting using TRIM37-specific antibodies (**Figure 1B**). We examined the status of a *TP53* mutation in GC cell lines by western blotting. The status of these TP53 mutations in various cell lines was positively associated with those in the database (http://p53.free.fr/index.html; W: wild-type TP53, M: mutant TP53). Note that, among TP53-mutated GC cell lines, KATO-III and HGC27 cells have a p53 gene deletion and a frameshift mutation, respectively.

### Immunohistochemical analysis of TRIM37 expression in primary tumors of gastric cancer

Because TRIM37 was overexpressed in almost all GC cell lines, it was hypothesized that TRIM37 would also be highly expressed in GC tissues and would be associated with carcinogenesis and malignant outcomes. We examined the prognostic and clinicpathological significance of TRIM37 expression in primary tumor samples of GC, based on the immunohistochemical staining pattern of this protein. TRIM37 was observed mainly in the cytoplasm of cancer cells. We classified 124 GC samples into either positive or negative groups according to the intensity of TRIM37 staining among tumor cells, as described in the *Materials and methods* section. In primary cases, TRIM37 expression was negative in the non-tumorous gastric mucosal cell population (**Figure 1C**). Kaplan-Meier survival estimates showed that TRIM37 immunoreactivity in tumor cells was significantly associated with overall survival according to the extent of each intensity score (**Figure 1D**). In the scores of intensities, the TRIM37 high-expression group, with a score ≥ 2 for tumor cells showing immunopositivity, showed significantly poorer overall survival (*P* = 0.0057, log-rank test) and relapse-free survival (*P* = 0.0288, log-rank test) compared with the low-expression group (**Figure 1D and 1E**).

### Association between TRIM37 protein abundance and clinicopathological characteristics in primary cases of gastric cancer

To test the hypothesis that TRIM37 protein abundance contributes to malignant features in GC, we assessed the expression of TRIM37 in primary GC tissues by immunohistochemistry. The relationship between the expression of TRIM37 and clinicopathological characteristics is summarized in **Table 1**. Protein expression of TRIM37 was significantly associated with being male, histopathological differentiation, venous invasion, lymphatic invasion, advanced pT and pN stage, and recurrence rate. A Cox proportional hazards regression analysis (**Table 2**) identified TRIM37 immunoreactivity in tumor cells as an independent factor that predicted a worse overall survival rate (hazard ratio 3.41; 95% confidence interval: 1.22-11.3), along with advanced pathological stage. Following gastrectomy, TRIM37 protein expression was significantly associated with recurrence (*P* =0.028), in particular lymphatic metastasis (*P* = 0.042) (**Table 3**).

### Suppression of cell proliferation by knockdown of TRIM37 and the effect of this knockdown according to TP53 mutation status

**Figure 2:**
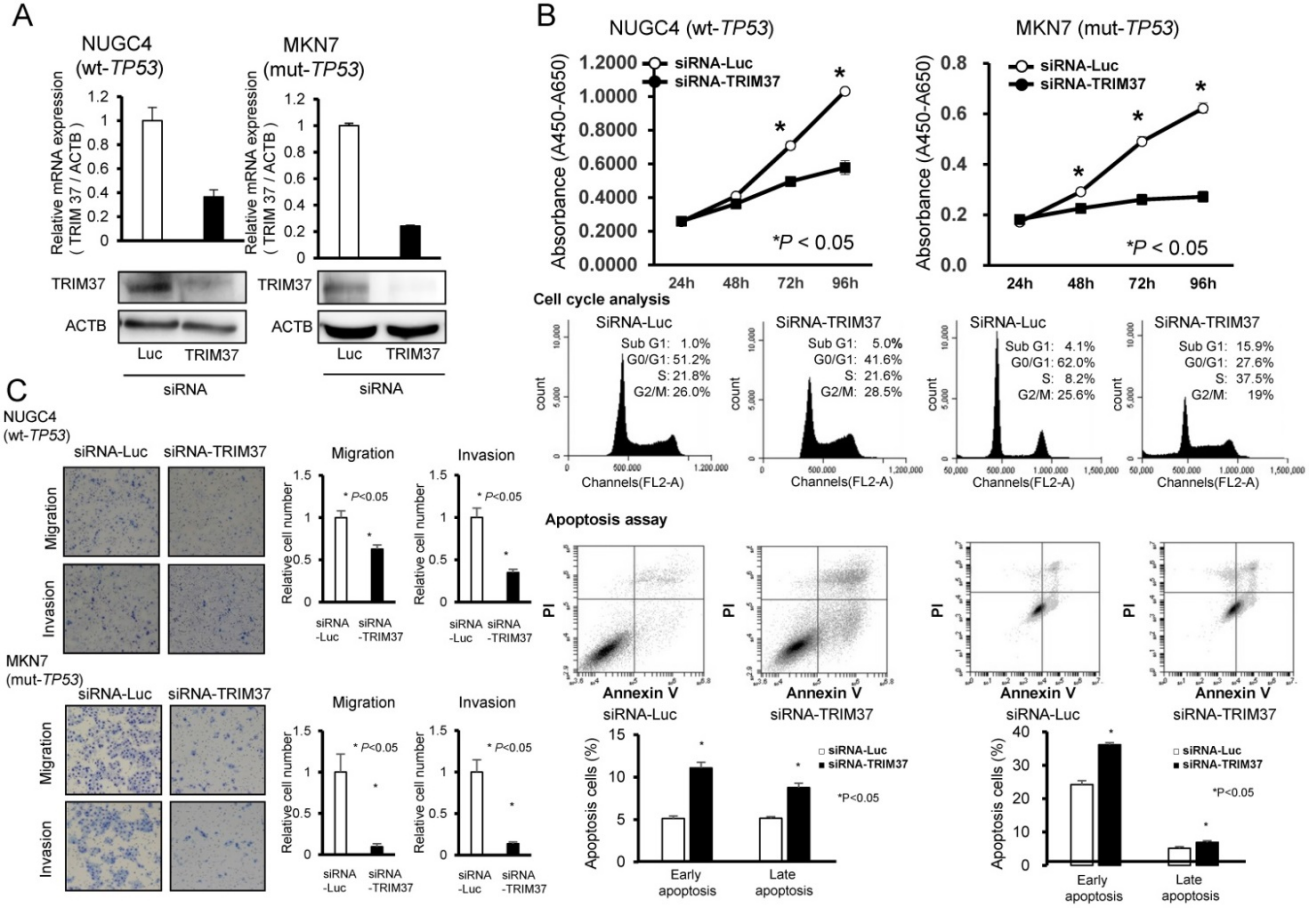
** Suppression of malignant behaviors in GC cells by knockdown of *TRIM37*. A.** Effects of TRIM37 knockdown by siRNA (siRNA-TRIM37) compared with those of control siRNA in NUGC4 (wild-type *TP53*) and MKN7 (mutant *TP53*) cell lines.** B.** Effects of knocking down endogenous TRIM37 on cell proliferation at the indicated times. The results shown are means ± SD (bars) for quadruplicate experiments. The Mann-Whitney U-test was used for the statistical analysis; * *P* < 0.05. (Upper-middle) Representative results of the population in each phase of the cell cycle in GC cells as assessed by FACS at 72 h post-treatment with siRNA. **C.** Migration and invasion of cells transfected with siRNA targeting *TRIM37* in NUGC4 and MKN7. The graphs show the means ± SD (bars) of *n* = 4. The Mann-Whitney U-test test was used for statistical analysis; *P* < 0.05 was considered to be statistically significant.

To gain insights into the potential role of *TRIM37* as an oncogene whose overexpression may be associated with gastric carcinogenesis, we first performed a cell-proliferation assay using siRNAs specific to *TRIM37* to investigate whether knockdown of TRIM37 would suppress the proliferation of GC cells that overexpress TRIM37. In the *p53* wild-type cell line, NUGC4, and the *p53* mutant cell line, MKN7, expression of the TRIM37 protein was efficiently knocked-down by the introduction of a *TRIM37*-specific siRNA (siRNA-*TRIM37*), and compared with a luciferase-specific siRNA (siRNA-*Luc*) as a negative control (**Figure 2A**). The proliferation of these cell lines was particularly suppressed following the knockdown of endogenous TRIM37 expression (**Figure 2B**).

### Cell cycle analysis and an apoptosis assay by the silencing of TRIM37 expression using fluorescence-activated cell sorting

To investigate the mechanisms by which the knockdown of TRIM37 suppressed cell proliferation, we performed a cell cycle analysis and an apoptosis assay. FACS analysis demonstrated that transfection of *TP53* wild-type NUGC4 cells with siRNA-*TRIM37* resulted in an accumulation of cells in G2/M phase compared with their transfection with control siRNA, while transfection of *TP53* mutant MKN7 with siRNA-*TRIM37* resulted in an accumulation of cells in the sub-G1 phase compared with transfection with control siRNA (**Figure 2B**).

Apoptotic cell analysis showed that transfection of *TP53* wild-type NUGC4 and *TP53* mutant MKN7 with siRNA-*TRIM37* increased early apoptosis (annexin V-positive/PI-negative) and late apoptosis (annexin V/PI-double positive), respectively, at 72 h post-transfection compared with transfection with control siRNA (**Figure 2B**). These findings suggest that the knockdown of TRIM37 overexpression induces cell apoptosis.

### Suppression of cell migration and invasion by the downregulation of TRIM37 expression

As shown in **Table 1**, protein expression of TRIM37 was significantly associated with both venous and lymphatic invasion in clinical samples. To confirm the relationship between TRIM37 and cell migration and invasion *in vitro*, transwell migration and invasion assays were performed. We examined the ability of *TP53* wild-type NUGC4 and *TP53* mutant MKN7 cells transfected with siRNA-*TRIM37* to move through pores under different conditions. An uncoated membrane was used for the migration assays, whereas a Matrigel-coated membrane was used for the invasion assays. As **Figures 2A and 2B** show, the number of siRNA-*TRIM37*-transfected NUGC4 and MKN7 cells that migrated into the lower chamber was significantly lower compared with siRNA-control-transfected cells under both conditions. These results suggest that the overexpression of TRIM37 may enhance the ability of GC cells to migrate and invade in both the *p53* wild-type and the *p53* mutant cell lines.

### Molecular mechanisms by which overexpression of TRIM37 contributes to malignant potential in gastric cancer cells

**Figure 3:**
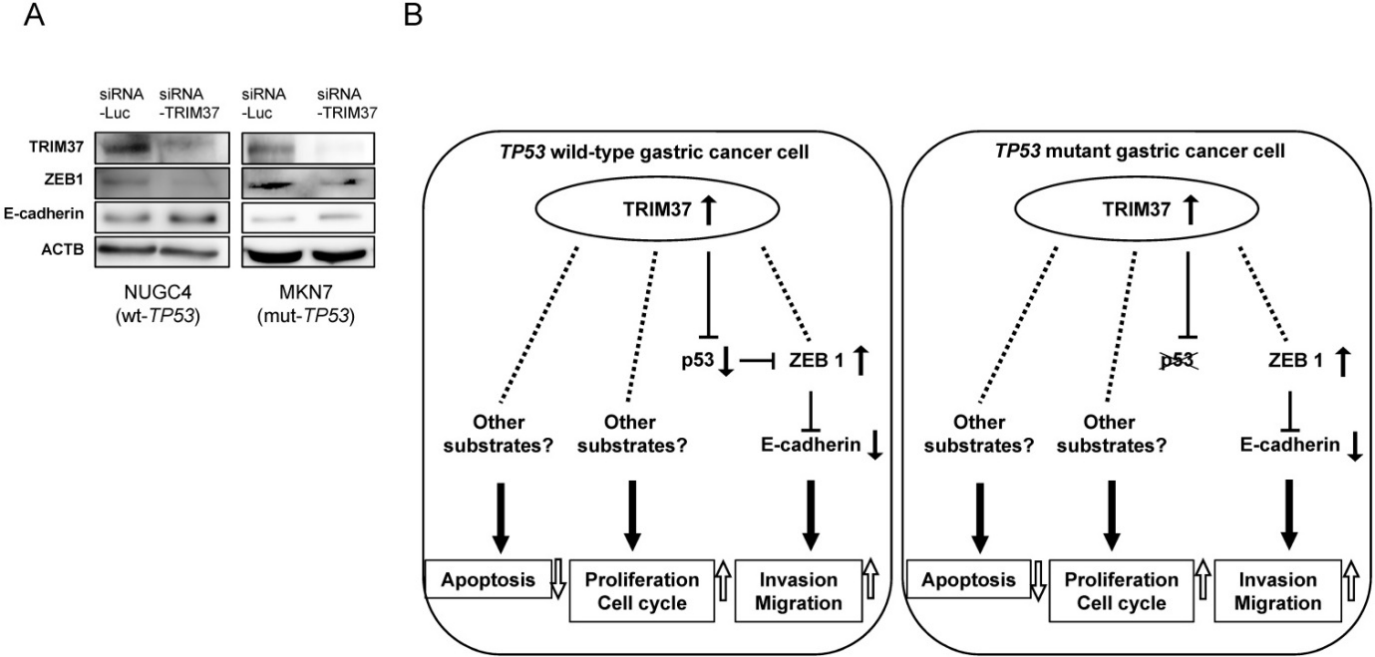
** Molecular mechanisms by which the overexpression of TRIM37 contributes to malignant potential in GC cells. A**. Knockdown of *TRIM37* by transfection with siRNA-*TRIM37* induced the production of ZEB1 and suppressed the production of E-cadherin in both NUGC4 and MKN7 cells. **B**. A hypothetical model of the overexpression/activation of TRIM37 in GC cells.

The most important hallmark of EMT is the loss of E-cadherin, which is mediated by ZEB1. 41 The knockdown of TRIM37 expression by transfection with siRNA-*TRIM37* suppressed the production of ZEB1 and induced the production of E-cadherin in both *TP53* wild-type and *TP53* mutant cells (NUGC4 and MKN7, **Figure 3A**). These results suggest that overexpression of TRIM37 induces cell migration and invasion via the loss of E-cadherin. **Figure 3B** shows a hypothetical model of the overexpression of TRIM37 in GC cells.

### Association between high levels of expression of TRIM37 and chemoresistance

**Figure 4:**
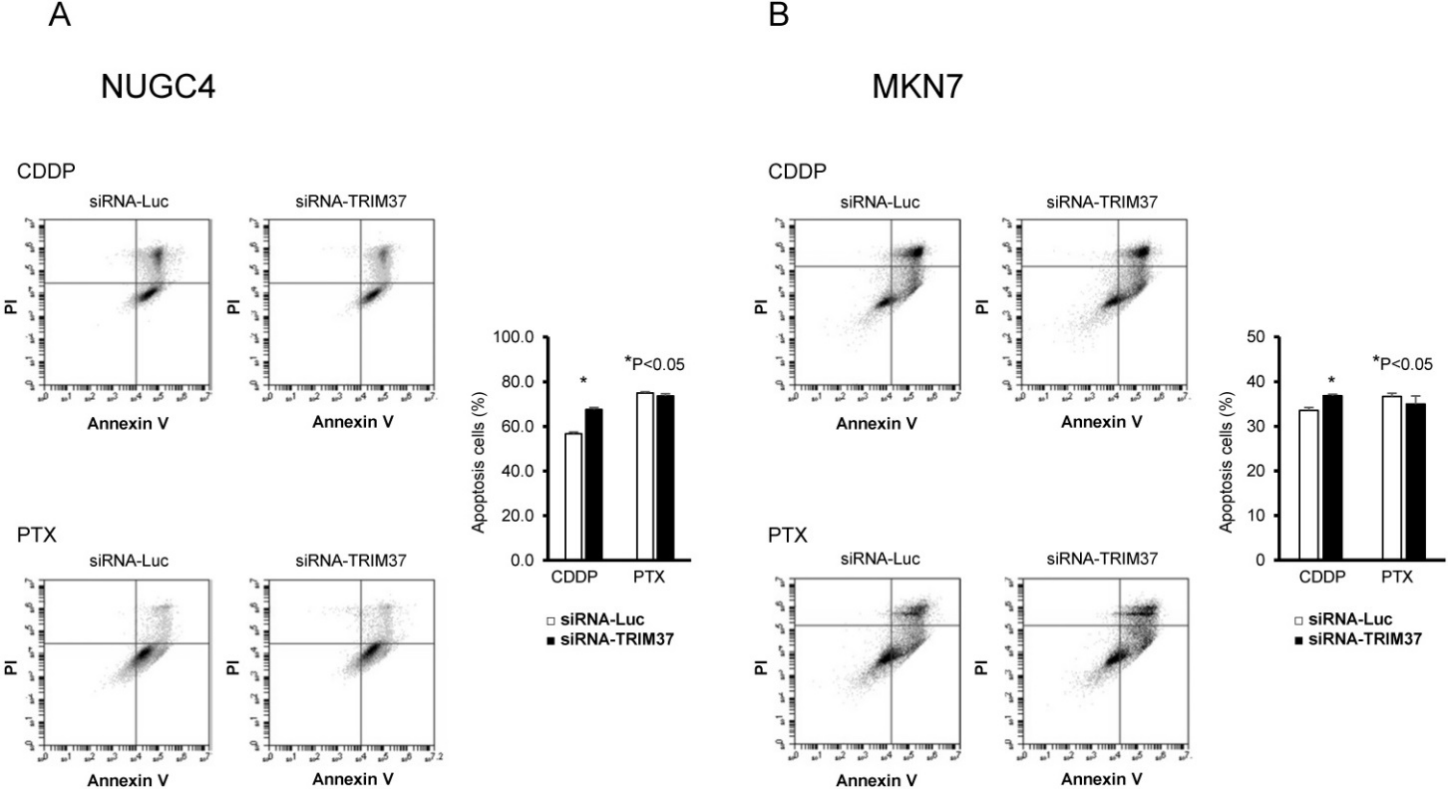
** Improvement of chemosensitivity by knockdown of TRIM37 in GC cells. A, B.** When treated with CDDP, transfection of *TP53* wild-type NUGC4 and *TP53* mutant MKN7 with siRNA-TRIM37 increased early apoptosis at 72 h post-transfection compared with transfection with control siRNA. When treated with paclitaxel (PTX), regardless of TRIM37 expression, it was not related to chemosensitivity in the *TP53* wild-type NUGC4 and *TP53* mutant MKN7.

Recently, it has been reported that high levels of expression of TRIM37 are associated with chemoresistance to CDDP in esophageal cancer and osteosarcoma 20, 25. Therefore, we examined whether TRIM37 was also associated with chemoresistance in GC. When treated with CDDP, transfection of *TP53* wild-type NUGC4 and *TP53* mutant MKN7 with siRNA-TRIM37 increased early apoptosis, at 72 h post-transfection, compared with transfection with control siRNA. However, when treated with PTX, chemosensitivity was not related to the status of TRIM37 expression in either the *TP53* wild-type NUGC4 or *TP53* mutant MKN7 (**Figure 4A and 4B**).

## Discussion

In this study, we demonstrated that TRIM37 is frequently overexpressed in primary GC cells and GC cell lines, and that this overexpression is significantly associated with malignant features of tumors and poor outcomes. Furthermore, the knockdown of TRIM37 inhibits cell proliferation, migration, invasion, and chemoresistance to CDDP. These findings suggest that TRIM37 plays a crucial role in tumor malignant potential through its overexpression and highlight its usefulness as a prognostic factor and potential therapeutic target in GC.

Recent studies identified that the mono-ubiquitination of histones is one of the most representative epigenetic markers and is intimately involved with Polycomb protein-mediated transcriptional gene-silencing. Polycomb complexes have crucial functions in cellular differentiation and development 42. Bhatnagar et al. reported that one of the main Polycomb protein complexes, Polycomb repressive complex 2 (PRC2), was associated with TRIM37. TRIM37, PRC2, and PRC1 are co-bound to tumor suppressive genes, resulting in their transcriptional silencing and oncogenic function 15. In our study, the immunoreactivity to the TRIM37 protein in each GC tissue was significantly associated with a worse clinical outcome in a multivariate analysis, even after stratification with other clinicopathological characteristics. This result suggests that immunoreactivity to TRIM37 may be a useful independent prognostic factor in patients with GC.

Chen et al. reported that TRIM37 promote cell invasion and metastasis in GC. In addition, our study revealed that TRIM37 promote cell proliferation and CDDP resistance 26. In our *in vitro* analyses, knockdown of TRIM37 overexpression induced G2/M cell-cycle arrest in wild-type *TP53* cells and G0/G1 cell-cycle arrest in mutant *TP53* cells. Balestra et al. demonstrated that TRIM37 acts to prevent centriolere duplication in HeLa cells 43 and Brigant et al. observed a significant increase in the percentage of cells in S phase in TRIM37-depleted chondrocyte cells 44. From these results, suppression of TRIM37 may arrest the cell cycle and suppress cell proliferation. We also investigated the molecular mechanisms affecting the malignant potential of tumor cells resulting from the overexpression of TRIM37. We found that the knockdown of TRIM37 suppressed cell migration and invasion in both wild-type and mutant *TP53* cells. Indeed, as shown in our immunohistochemical analysis, the overexpression of *TRIM37* is related to the presence of lymphatic and venous invasion. Moreover, patients with GC that showed high levels of TRIM37 expression GC had worse prognostic outcomes and a higher incidence of lymphatic recurrence (**Table 3**). Chen et al. also reported that TRIM37 was significantly associated with lymph node metastasis 26. Thus, our *in vitro* findings supported the *in vivo* results of our immunohistochemical analysis and strongly suggested that TRIM37 plays a pivotal role in the malignant potential of GC.

Recent studies regarding chemotherapy identified that the downregulation of TRIM37 in esophageal cancer and osteosarcoma cells presented CDDP-induced cell apoptosis 20, 25. CDDP is a key chemotherapy drug for locally advanced or metastatic carcinoma of gastric 45, 46. PTX is another important chemotherapy drug recommended for use in second-line treatment by the Japanese guidelines for the treatment of GC 47, 48. Therefore, we wished to verify the effect of TRIM37 on chemoresistance to CDDP or PTX in GC. We found that knockdown of TRIM37 was associated with chemoresistance to CDDP, but not with chemoresistance to PTX. These findings suggest that TRIM37 may be a key molecule for predicting chemoresistance and improving chemosensitivity to CDDP in prospective GC patients who exhibit overexpression of TRIM37. Also, PTX may be a preferable alternative treatment option for patients with high levels of TRIM37. The detailed mechanisms of chemoresistance and the effects on other chemotherapeutic agents are currently being investigated.

In conclusion, we clearly demonstrated the frequent overexpression of the TRIM37 protein and its prognostic value in patients with GC. Although studies involving larger cohorts will be necessary to validate these findings before moving on to explore their application to clinical settings, our results provide evidence that TRIM37 could be a crucial molecular marker for determining the malignant properties of GC cells and also that it could be a target for molecular therapy in patients with GC.

## Supplementary Material

Supplementary figure S1.

## Figures and Tables

**Table 1:** Association between clinicopathological characteristics and TRIM37 expression

Characteristics	n	TRIM37 immunoreactivity				*P* value ^a^
		High	(%)	Low	(%)	
Total	124	76		48		
**Gender**						**0.017**
Male	84	58	76	26	54	
Female	40	18	24	22	46	
**Age (years)**						0.581
< 65	65	38	50	27	56	
≥ 65	59	38	50	21	44	
**Location**						0.836
Upper	15	10	13	5	10	
Middle	66	39	51	27	56	
Lower	43	27	36	16	33	
**Histological grade**						**<0.001**
Differentiated	56	46	61	10	21	
Undifferentiated	68	30	39	38	79	
**Venous invasion**						**0.004**
Negative	87	46	61	41	85	
Positive	37	30	39	7	15	
**Lymphatic invasion**						**0.009**
Negative	66	33	43	33	69	
Positive	58	43	57	15	31	
***TNM classification***						
**pT categories**						**0.013**
T1	68	35	46	33	69	
T2	9	7	9	2	4	
T3	20	18	24	2	4	
T4	27	16	21	11	23	
**pN categories**						**0.016**
N0	77	41	54	36	75	
N1	18	12	16	6	13	
N2	8	4	5	4	8	
N3	21	19	25	2	4	
**pStage**						0.103
I	70	39	51	31	65	
II	20	11	14	9	19	
III	34	26	34	8	17	
**Recurrence**						**0.046**
Absent	96	54	71	42	88	
Present	28	22	29	6	13	

**Note:** Statistically significant values are in bold type; ^a^ *P* values are from χ^2^ or Fisher's exact test and were statistically significant at < 0.05.

**Table 2:** Cox proportional hazard regression analysis for overall survival

Variable	Univariate ^a^	Multivariate ^b^		
	*P*-value ^c^	HR	95%CI	*P*-value ^c^
**Gender**				
Male vs. Female	0.633		-	
**Age (years)**				
≤ 60 vs. < 60	**0.045**		-	
**Histological grade**				
Undifferentiated vs. Differentiated	0.842		-	
**Venous invasion**				
Positive vs. Negative	**< 0.001**	3.14	1.15-9.46	**0.024**
**Lymphatic invasion**				
Positive vs. Negative	**< 0.001**			
**pStage**				
Stage III vs. I / II	**< 0.001**	11.3	3.66-44.5	**< 0.001**
**TRIM37 expression ^d^**				
High vs. Low	**0.014**	3.41	1.22-11.3	**0.018**

Statistically significant values are in boldface type; Abbreviations: CI = confidence interval; HR = hazard ratio. ^a^ Kaplan and Meier method, and the statistical significance was determined by log-rank test. ^b^ Multivariate survival analysis was performed using Cox's proportional hazard model. ^c^ *P*-values were from two-sided tests and were statistically significant at <0.05. ^d^ TRIM37 expression was evaluated by immunohistochemical analysis as described in Materials and Methods.

**Table 3:** Association between the expression of TRIM37 and disease recurrence in GC patients with gastrectomy

	n	TRIM37 expression		*P-value* ^a^
		High	Low	
Number of patients	124	76	48	
Total of recurrence	28	22 (29%)	6 (13%)	**0.046**
Liver recurrence	4	4 (5%)	0 (0%)	0.158
Lymphatic recurrence	7	7 (9%)	0 (0%)	**0.042**
Peritoneal recurrence	12	7 (9%)	5 (10%)	1.000
Local recurrence	3	2 (3%)	1 (2%)	1.000

^a^Chi-square test or Fischer's exact test; **Note:** Significant values are in bold.

## Abbreviations

GC: Gastric cancer
TRIM37: Tripartite Motif Containing 37
CDDP: Cisplatin
PTX: Paclitaxel
