# Supplementary material for: TRIM37 contributes to malignant outcomes and CDDP resistance in gastric cancer
Source: J Cancer. 2021 Jan 1;12(2):316–25. doi: 10.7150/jca.47577 (PMC7739001; doi:10.7150/jca.47577)
Supplement: Supplementary file 1 — Supplementary figure S1. [file jcav12p0316s1.pdf]

Figure legends

Figure S1.

A Overexpression of TRIM37 in cells

Expression of TRIM37 mRNA in GC cell lines compared with cells from healthy organs plus the fibroblast cell line WI-38.

B Cell cycle analysis by the silencing of TRIM37 expression

Transfection of *TP53* wild-type NUGC4 cells with siRNA-*TRIM37* resulted in an accumulation of cells in G2/M phase compared with their transfection with control siRNA, however transfection of *TP53* mutant MKN7 with siRNA-*TRIM37* resulted in an accumulation of cells in the sub-G1 phase compared with transfection with control siRNA

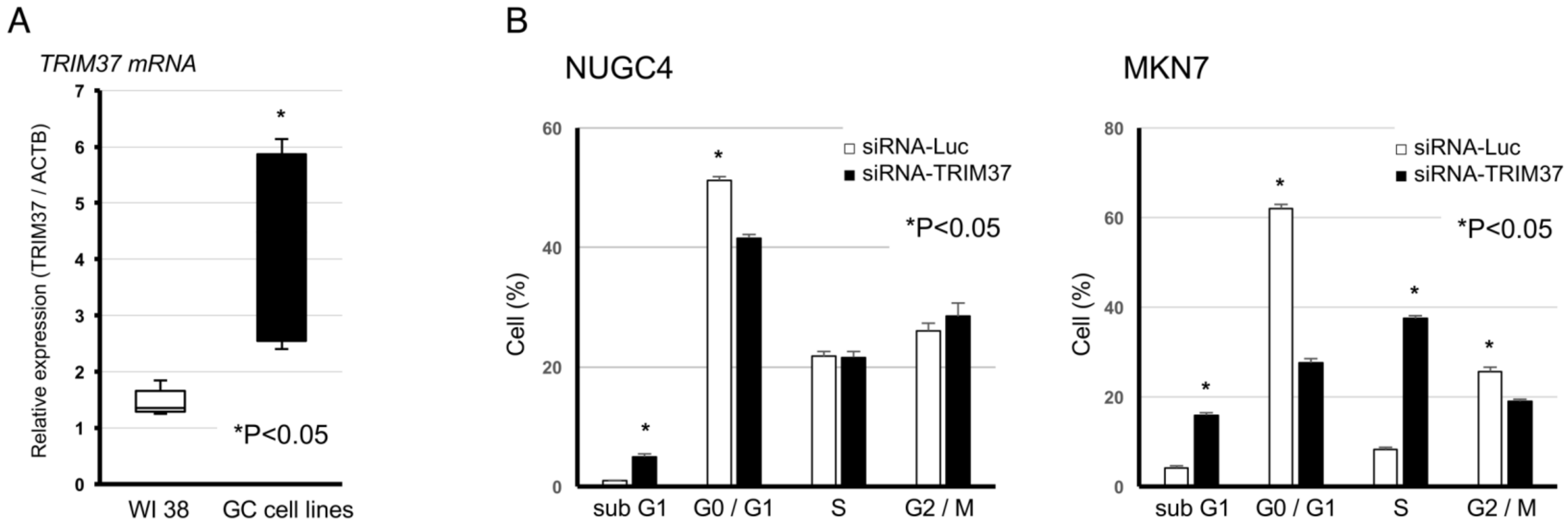

A
TRIM37 mRNA
Relative expression (TRIM37 / ACTB)
7
6
5
4
3
2
1
0
*
*P<0.05
WI 38
GC cell lines
B
NUGC4
Cell (%)
60
40
20
0
siRNA-Luc
siRNA-TRIM37
*P<0.05
sub G1
G0 / G1
S
G2 / M
MKN7
Cell (%)
80
60
40
20
0
siRNA-Luc
siRNA-TRIM37
*P<0.05
sub G1
G0 / G1
S
G2 / M
